# Aurantoside C Targets and Induces Apoptosis in Triple Negative Breast Cancer Cells

**DOI:** 10.3390/md16100361

**Published:** 2018-10-01

**Authors:** Sumi Shrestha, Anabel Sorolla, Jane Fromont, Pilar Blancafort, Gavin R. Flematti

**Affiliations:** 1 School of Molecular Sciences, The University of Western Australia, Crawley, WA 6009, Australia; sumi.shrestha@research.uwa.edu.au; 2 Cancer Epigenetics, Harry Perkins Institute of Medical Research, QEII Medical Centre and Centre for Medical Research, The University of Western Australia, Crawley, WA 6009, Australia; anabel.sorollabardaji@perkins.uwa.edu.au; 3 Western Australian Museum, Welshpool, WA 6106, Australia; jane.fromont@museum.wa.gov.au

**Keywords:** triple negative breast cancer, aurantoside C, marine sponge, apoptosis, cell cycle analysis

## Abstract

Triple negative breast cancer (TNBC) is a subtype of breast cancers that currently lacks effective targeted therapy. In this study, we found that aurantoside C (C828), isolated from the marine sponge *Manihinea* *lynbeazleyae* collected from Western Australia, exhibited higher cytotoxic activities in TNBC cells compared with non-TNBC (luminal and normal-like) cells. The cytotoxic effect of C828 was associated to the accumulation of cell at S-phase, resulting in the decline of cyclin D1, cyclin E1, CDK4, and CDK6, and an increase in p21. We also found that C828 inhibited the phosphorylation of Akt/mTOR and NF-kB pathways and increased the phosphorylation of p38 MAPK and SAPK/JNK pathways, leading to apoptosis in TNBC cells. These effects of C828 were not observed in non-TNBC cells at the concentrations that were cytotoxic to TNBC cells. When compared to the cytotoxic effect with the chemotherapeutic drugs doxorubicin and cisplatin, C828 was found to be 20 times and 35 times more potent than doxorubicin and cisplatin, respectively. These results indicate that C828 could be a promising lead for developing new anticancer agents that target TNBC cells.

## 1. Introduction

Breast cancer is the most prevalent malignancy in women worldwide [1]. Based on gene expression profiles, breast cancers have been grouped into five different subtypes, namely luminal A, luminal B, human epidermal growth factor receptor 2 (HER2)-enriched, basal-like, and normal like subtypes [2]. The luminal A, luminal B, and HER2 tumors are easily identified due to the presence of biomarkers and can be treated with the most appropriate chemotherapeutic agents, which typically involve hormonal and trastuzumab-based therapies [3]. In contrast, basal-like is one of the most aggressive types of breast cancer lacking estrogen receptor (ER), progesterone receptor (PR) and HER2, and possessing a high expression of epithelial-to-mesenchymal transition markers as well as enrichment in stem cell-like features [2,4,5,6]. The vast majority of tumors lacking the ER, PR, and HER2 receptors (triple negative) belong to the basal-like subtype and also to the recently found claudin-low subtype [7]. TNBC comprise approximately 15% of all breast cancers [8] that cannot be treated from hormonal and trastuzumab-based therapies, leaving TNBC patients with fewer and unspecific treatment options [9]. Current chemotherapeutic options are non selective for the treatment of TNBC. Thus, there is a pressing need for the development of more selective and potent therapeutic strategies to combat such malignancies.

TNBC cells are characterized by the increase in phosphorylation of the phosphatidylinositol-3-kinase (PI3-K)/Akt/mammalian target of rapamycin (mTOR) signaling pathways [10,11,12]. Akt is the downstream effector of PI3K and mTOR is the downstream effector of Akt pathways [12]. Full activation of Akt requires dual phosphorylation of Ser^473^ by mTOR and on Thr^308^ by PDK1 [13]. Moreover, mTOR regulates the synthesis of proteins that are responsible for the growth and survival of cancer cells [14]. The activated PI3K/Akt/mTOR pathway favors metastatic progression and chemotherapy/radiotherapy resistance [15]. Therefore, inhibition of these pathways can overcome resistance to chemotherapy in TNBC and block aggressive phenotypes in these cancers [16]. Similarly, the NF-κB pathway is a major regulator of cell proliferation, survival, differentiation, and immune response and its activation also confers cancer cells with drug resistance mechanisms [17,18]. NF-κB pathway is found to be activated in TNBC and its inhibition suppressed proliferation in TNBC cells [19]. It was also found that the upregulation of Akt and NF-κB pathways inhibits apoptosis [18,20]. Moreover, the upregulation of Akt is related to the activation of cyclin D1, which is further responsible for proliferation in TNBC [21]. Therefore, inhibition of these pathways can be regarded as an attractive target for treating TNBC.

Another signaling pathway involved in TNBC is the MAPK signaling pathway. The MAPK pathway causes various cellular processes such as cell survival and differentiation [22]. The aberrant and overexpression of MAPK in TNBC may contribute to the progression of cancer cells, leading to uncontrolled proliferation and resistance to apoptosis [23]. MAPK is classified into three major groups, namely, extracellular signal-regulated kinases (ERKs), stress-induced protein kinase (SAPK)/JNK, and p38 MAPK [24]. The significance of MAPK signaling pathways in sensitivity or resistance of chemotherapeutic drugs in cancer cells is still not clearly understood [24]. Studies have shown that the activation of ERK causes resistance to chemotherapeutic drugs 5-fluorouracil and doxorubicin in pancreatic cancer cells [24]. On the other hand, SAPK/JNK and p38 MAPK are responsible for stress-dependent apoptosis induced by various factors including chemotherapeutic drugs [25]. As for example, the activation of SAPK/JNK and p38 MAPK decreases resistance of chemotherapeutic drug cisplatin thus, activating FasL-mediated apoptosis in ovarian cancer cells [26,27]. Thus, the activation of SAPK/JNK and p38 MAPK pathways could be a desirable feature for any therapeutic agent targeting TNBC.

Natural products have traditionally been a rich source of novel lead compounds in drug discovery [28,29]. In particular, compounds isolated from marine organisms have been a source of anticancer agents [30,31]. Currently, there are three marketed drugs for cancer from marine organisms namely, cytarabine C (for treatment of acute myelogenous) from the sponge *Tectitethya crypta* [32], eribulin mesylate (used to treat metastatic breast cancer), a synthetic analogue of halichondrin B, isolated from the sponge *Halichondria okadai* [33], and trabectedin (to treat advanced or metastatic soft-tissue sarcoma or ovarian cancer) isolated from the marine tunicate *Ecteinascidia turbinata* [34]. In addition, there are also other compounds derived from marine natural products undergoing clinical trials such as Plitidepsin, from the marine tunicate *Aplidium albicans* for treatment of multiple myeloma, bryostatin-1 isolated from the marine bryozoan *Bugula neritina* for treating metastatic colorectal cancer, and zalypsis, a synthetic derivative of jorumycin, isolated from the nudibranch *Jorunna funebris* for treatment of urothelial carcinoma [34]. Among various novel and bioactive compounds isolated, 75% of them have been isolated from marine sponges and most of them show cytoxicity in cancer cells, hence, marine sponges have been identified as the most promising source of anticancer compounds [35,36].

Recently, we reported the isolation of crambescidin 800 (C800) from the marine sponge *Monanchora viridis* collected off the coast of Western Australia (WA) and showed it had cytotoxic activity in TNBC cells [37]. In the current work, we showed that the crude extract of the sponge *Manihinea lynbeazleyae* inhibited the cell viability of TNBC cells leaving the non-TNBC (luminal and normal-like) cells unaffected. Through bioassay-guided fractionation of the sponge *M. lynbeazleyae*, we isolated aurantoside C (C828) as the active compound. Of note, C828 possesses high cytotoxic activity in the very low micro-molar range of concentrations in TNBC cells. In particular, we found that C828 exhibited cytotoxic activity in TNBC cells over non-TNBC cells through the accumulation of the cells at the S-phase, decline in the cyclins and cyclin dependent kinases and increase in the CDK inhibitor, p21. We also observed that C828 inhibited the phosphorylation of Akt/mTOR and NF-kB signaling pathways and at the same time activated phosphorylation of p38 MAPK and SAPK/JNK signaling pathways, ultimately leading TNBC cells to apoptotic cell death, while non-TNBC cells remained unaffected. Altogether, these findings reveal new knowledge in the mode of action of aurantoside C (C828) in TNBC cells and also demonstrate the efficacy of C828 as a novel promising natural agent for targeting TNBC. 

## 2. Results

### 2.1. Screening the Crude Extract of the Sponge Manihinea Lynbeazleyae

**Figure 1:**
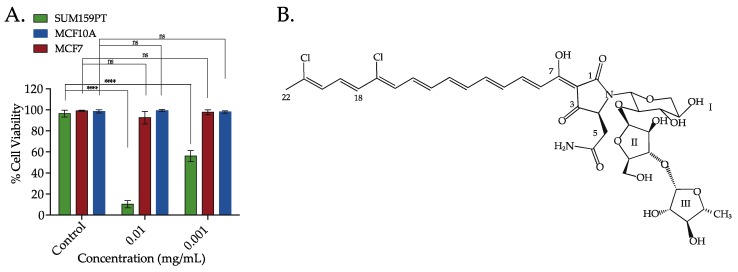
The crude extracts of the sponge *Manihinea lynbeazleyae* collected off the coast of Western Australia was screened for cytotoxic effect in TNBC and non-TNBC cells and the chemical structure of the bioactive compound isolated. (**A**) Percentage of cell viability in TNBC SUM159PT cells, and non-TNBC MCF7 and MCF10A cells after treatment with crude solvent extracts of sponge *M. lynbeazleyae* for 24 h. CellTiter-Glo^®^ was used to measure cell viability. Three independent experiments were performed, each of them done in triplicates. One way ANOVA with Tukey’s posthoc test was used for statistical analysis **** *p* < 0.0001, and ns = not significant. (**B**) Chemical structure of Aurantoside C (C828) isolated as the bioactive compound.

The cytotoxic activity of the crude extract of the sponge *M. lynbeazleyae* was screened in TNBC claudin-low cell line (SUM159PT) and non-TNBC luminal and normal-like epithelial cell lines (MCF7 and MCF10A, respectively). We used 0.1% DMSO that was diluted in cell media as vehicle control. Percentages of cell viability were calculated and were relative to the vehicle control. The crude solvent extracts of *M*. *lynbeazleyae* at 0.01 mg extract/mL and 0.001 mg extract/mL reduced the percentage of cell viability to 10% and 60%, respectively, in SUM159PT cells compared to vehicle control cells. At the same concentrations, the percentage of cell viability of luminal and normal-like cells was approximately 90%. These results show that the crude extract of *M*. *lynbeazleyae* significantly reduced the percentage of cell viability in TNBC cells whereas very minor cytotoxicity was observed in non-TNBC cells (Figure 1A). As the crude extract was active in SUM159PT cells, further bioassay-guided fractionation and purification of the extract to isolate the active compound was performed in these cells.

### 2.2. Bioassay-Guided Fractionation, Isolation and Characterisation of Aurantoside C

For the isolation of the active compound, the crude extract was separated using flash silica chromatography with gradient elution starting from 100% hexanes to 100% ethyl acetate then to 100% methanol to give six different fractions. Testing of the fractions in SUM159PT cells showed that the 100% methanol fraction was the most active. The active fraction was separated further using high-pressure liquid chromatography (HPLC) with an isocratic mobile phase of 55% (*v*/*v*) acetonitrile/water (+0.1% TFA). A total of nine fractions were collected over 30 min and tested for cell viability in SUM159PT cells. The fraction collected between retention times 17–19 min was the most active and corresponded with a major peak detected at 490 nm. This fraction was collected, concentrated under reduced pressure to afford an amorphous bright red solid consisting of mainly one compound (>95%). Analysis by high-resolution mass spectrometry (HRMS) indicated a molecular ion [M + H]^+^ at *m*/*z* 829.6216 which corresponded to a molecular formulae of C_37_H_46_N_2_O_15_Cl_2_. Based on literature search and 1D and 2D NMR data, together with a literature comparison that confirmed the isolated compound as Aurantoside C (C828) (Figure 1B). Aurantoside C was previously isolated from the sponge *Homophymia conferta* (now known as *Manihinea conferta*) [38] but the cytotoxic activity of C828 has not yet been studied.

### 2.3. Aurantoside C Shows Preferential Cytotoxicity in TNBC Cells Compared to Non-TNBC Cells

**Figure 2:**
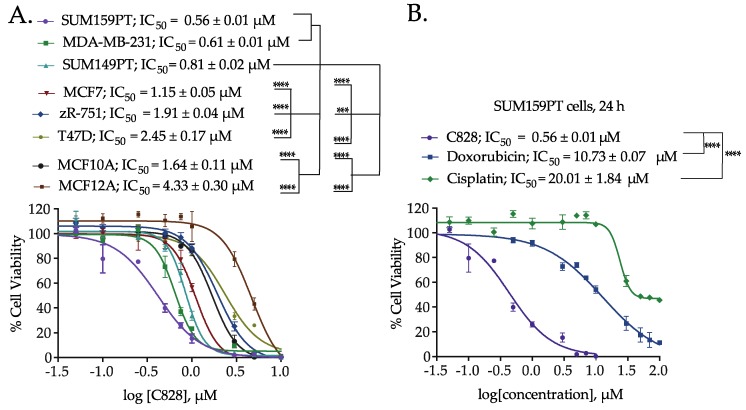
Comparison of the cell viability of Aurantoside C (C828) in TNBC cells and non-TNBC (luminal and normal-like) cells. (**A**) Dose response curve showing the cytotoxic effect of C828 after 24 h of treatment in a range of human TNBC, luminal and normal-like breast epithelial cells. (**B**) The dose-response curves of C828 compared with chemotherapeutic drugs, cisplatin and doxorubicin on SUM159PT cells after 24 h of treatment. Three independent experiments were performed, each of them done in triplicates. The graphs plotted are the representative graphs and the results represent IC_50_ ± SD from three independent experiments. One way ANOVA with Tukey’s post hoc test was used for statistical analysis **** *p* < 0.0001, and *** *p* < 0.001.

Since the *M. lynbeazleyae* crude extract showed increased cytotoxicity in TNBC cells compared to non-TNBC cells, the cytotoxic activity of C828 in these cells was compared. The percentage of cell viability after treatment with C828 in a larger panel of TNBC cells that includes SUM159PT and MDA-MB-231 (claudin-low), and SUM149PT (basal-like) along with non-TNBC cells such as MCF7, ZR-75-1 and T47D (luminal cells), and MCF10A and MCF12A (normal-like breast epithelial cells) were investigated. We observed that C828 was more effective in reducing cell viability in SUM159PT, MDA-MB-231 and SUM149 with the IC_50_ values of 0.56 ± 0.01 µM, 0.61 ± 0.01 µM, and 0.81 ± 0.02 µM, respectively. In contrast, C828 showed less efficiency in inducing cell death in the non-TNBC cells MCF7, ZR-75-1 and T47D with the IC_50_ values of 1.15 ± 0.05 µM, 1.91 ± 0.04 µM and 2.45 ± 0.17 µM, respectively. As for the normal-like cells, MCF10A and MCF12A, the IC_50_ values obtained were 1.64 ± 0.11 µM and 4.33 ± 0.30 µM, respectively (Figure 2A). Therefore, the effect of C828 in TNBC cells was significantly higher than in non-TNBC cells. As the effect of C828 in SUM159PT cells (TNBC), compared with MCF7 and MCF10A (non-TNBC), was more pronounced, further tests were carried out in SUM159PT cells (TNBC), while MCF7 and MCF10A cells were used as control cell lines.

No significant change in IC_50_ was seen when SUM159PT, MCF7, and MCF10A cells were treated with C828 for extended time periods (48 h and 72 h), as shown in supplementary Figure S3A–C. We also found that in the TNBC cells tested, C828 was more potent than our previously reported compound C800 when treated for 24 h, as shown in Table 1 [37]. 

### 2.4. Aurantoside C is More Efficient in Inducing Cell Death in TNBC Cells than Commonly Used Chemotherapeutic Drugs

Doxorubicin and cisplatin are drugs currently used in chemotherapy to treat TNBC patients [39,40,41]. The percentage of cell viability when treated with C828 for 24 h was compared with that of doxorubicin and cisplatin in SUM159PT cells. The IC_50_ of C828 in SUM159PT cells was 0.56 ± 0.01 µM, whereas that of doxorubicin and cisplatin was found to be 10.73 ± 0.07 µM and 20.01 ± 1.84 µM, respectively (Figure 2B). Intriguingly, this means that C828 is 20 and 35 times more potent than doxorubicin and cisplatin respectively in TNBC cells.

### 2.5. Aurantoside C Induces Accumulation of Cells at S-phase in TNBC Cells 

**Figure 3:**
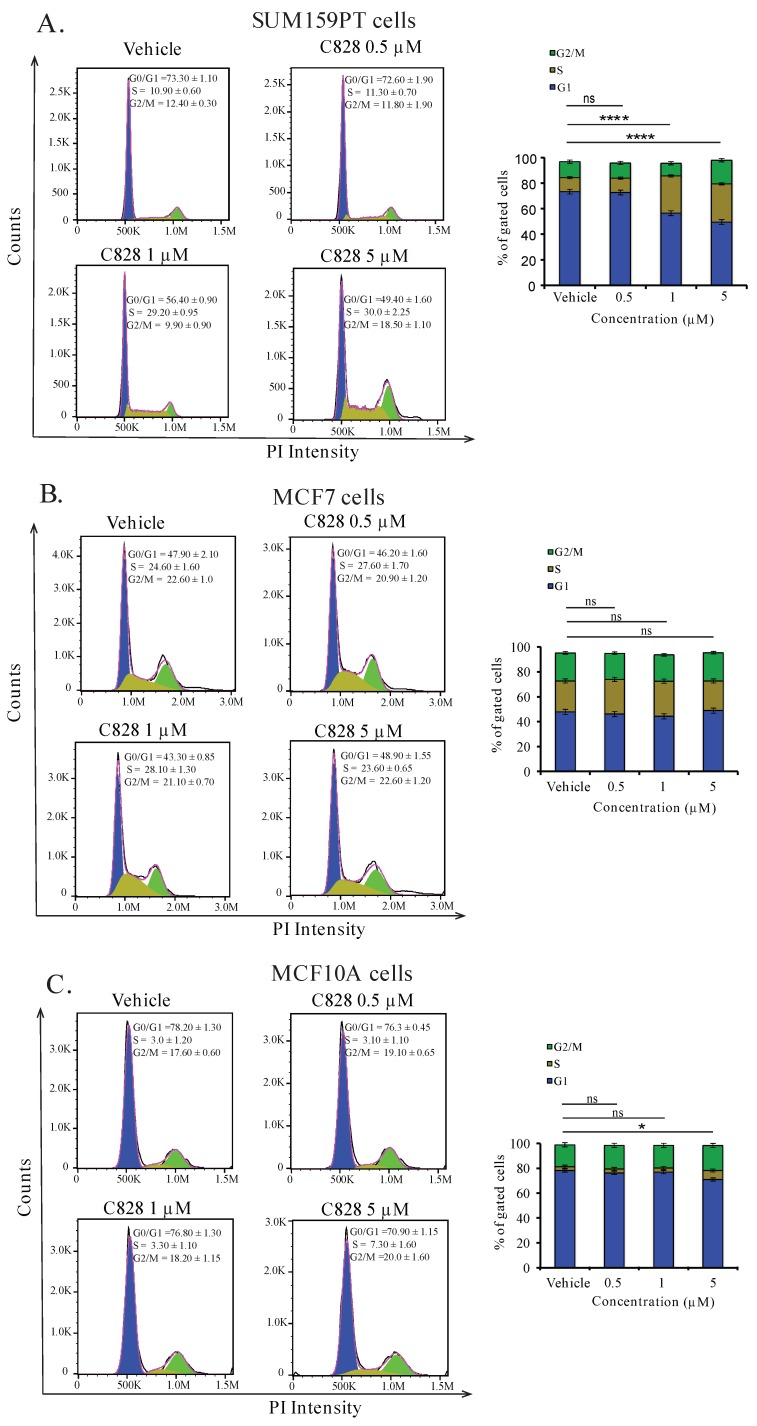
C828 increase DNA content at S-phase of the cycle in TNBC cell lines but not in non-TNBC cells. Effect of C828 on cell cycle distribution in (**A**) SUM159PT cells, (**B**) MCF7, and (**C**) MCF10A cells. Cells were treated with vehicle, 1 μM and 5 μM of C828 for 24 h. 0.1% DMSO diluted in media was used as a vehicle control. The distribution of cells in different phases of the cycle was measured by flow cytometry and data analyzed by FlowJo. Three independent experiments were performed, each of them done in triplicates. The fractions of cells in each phase of the cycle is shown in bar diagram as the mean ± S.D. One way ANOVA, with Tukey’s post hoc test was used for statistical analysis **** *p* < 0.0001, * *p* < 0.1 and ns = not significant.

As the distribution of cells after treating with drugs in different phases of cell cycle can reveal the mechanism involved, we next examined the cell cycle pattern in SUM159PT, MCF7, and MCF10A cells after treatment with C828. After treatment with 0.5, 1, and 5 µM of C828 for 24 h, DNA contained in the cells were stained with PI and the cell cycle distribution pattern was analyzed using flow cytometry [42,43,44]. Treatment of cells with 0.1% DMSO was used as vehicle control. Flow cytometry data revealed that 24 h of exposure of SUM159PT cells to C828 showed a significant rise in DNA content in S-phase of the cycle from 10.9% in vehicle control to 29.2% and 30.0% when treated with one µM and five µM C828 treated cells, respectively (Figure 3A). In contrast, the DNA content in the cells in S-phase remained almost constant at 24.6%, 28.1%, and 23.6% in the non-TNBC cell line MCF7 when treated with 0.5 µM, one µM, and five µM C828 with respect to 24.6% in vehicle control (Figure 3B). Similarly, in MCF10A cells, the DNA content in S-phase remained constant at 3.0% and 3.3% in 0.5 µM and one µM with respect to vehicle control whereas increased slightly to 7.3% when treated with 5 µM C828. However, the increase was not significant (Figure 3C). Thus, this result shows that C828 significantly caused accumulation of cells at the S-phase in TNBC cells, while no significant effect in non-TNBC cells were observed. This result together with the differential effect in cell viability of TNBC cells versus non-TNBC cells suggest C828 being more sensitive towards cells presenting a more aggressive cancer phenotype. 

### 2.6. Aurantoside C Regulates Cell Cycle Related Proteins in TNBC Cells

**Figure 4:**
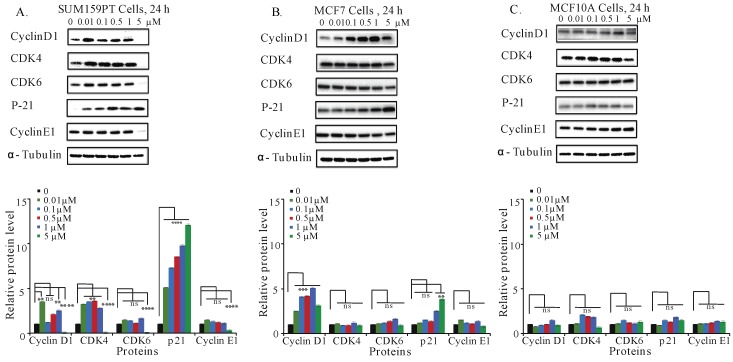
C828 inhibits cell cycle related proteins in TNBC cell lines. Effect of C828 on cell cycle related proteins in (**A**) SUM159PT, (**B**) MCF7, and (**C**) MCF10A cells. Cells were treated with 0.01, 0.1, 0.5, 1 and 5 μM of C828 for 24 h. Cells treated with 0.1% DMSO diluted in media were used as a vehicle control. Whole cell lysates were isolated, quantification of proteins was done and immunoblotted with antibodies specific for cyclin D1, CDK4, CDK6, cyclinE1, CDK2, and p21. α-tubulin was used as a loading control for each experiment. ImageJ was used to quantify each blot. The blots were normalized against tubulin and then against control protein (when C828 was not added). Two independent experiments were performed. The histogram below each Western blot represents the average normalized values of the band from each experiment. One way ANOVA with Tukey’s posthoc test was used for statistical analysis **** *p* < 0.0001, *** *p* < 0.001, ** *p* < 0.01, and ns = not significant.

The accumulation of the cells at S-phase of the cycle induced by C828 in SUM159PT cells led us to further investigate the regulation of cell cycle related proteins in SUM159PT, MCF7, and MCF10A cells. Cyclins and cyclin dependent kinases (CDK), namely, cyclin D1, CDK4, and CDK6 regulate cell cycle progression, whereas cyclin dependent kinase inhibitor (CDKI), namely p21 deregulates the cell cycle [45,46]. It has also been reported that the overexpression of p21 inhibits proliferation of cancer cells causing cell cycle arrest [47,48]. Treatment of cells with various concentrations (0, 0.01, 0.1, 0.5, one, and five µM) of C828 was done for 24 h. Changes in different proteins were evaluated by immunoblotting with respective primary antibodies. In SUM159PT cells, five µM of C828 inhibited the protein expression of cyclin D1, CDK4, CDK6, and cyclin E1 (Figure 4A). The expression of p21 was increased for all concentrations tested in SUM159PT cells (Figure 4A). In contrast, the expressions of cyclin D1, CDK4, CDK6, cyclin E1 and p21 in MCF7 and MCF10A cells remained unchanged (Figure 4B,C). Thus, C828 regulated cyclins and CDKI in TNBC cells, while no change was observed in non-TNBC cells. These results suggest that C828 prevents cell cycle progression in TNBC cells through the activation of CDKI, p21.

### 2.7. Aurantoside C Inhibits Phosphorylation of Akt/mTOR and NF-κB Signaling Pathways in TNBC Cells

**Figure 5:**
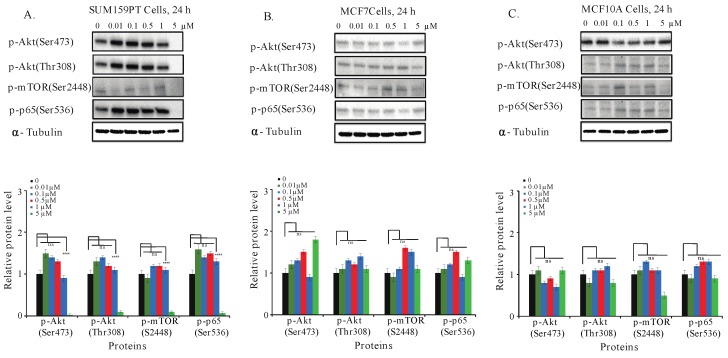
Effect of C828 in the phosphorylation of Akt/mTOR and NF-κB pathways in TNBC and non-TNBC cells. (**A**) SUM159PT cells, (**B**) MCF7 cells, and (**C**) MCF10A cells were treated with 0, 0.01, 0.1, 0.5, 1, and 5 μM of C828 for 24 h. Whole cell lysates were isolated, quantification of proteins was done and immunoblotted with antibodies specific for p-Akt (Ser473), p-Akt (Thr308), p-mTOR (Ser2448) and p-p65 (Ser536). α-tubulin was used as a loading control for each experiment. ImageJ was used to quantify each blot. The blots were normalized against tubulin and then against control protein (when C828 was not added). Two independent experiments were performed. The histogram below each western blot represents the average normalized values of the band from each experiment. One way ANOVA with Tukey’s post hoc test was used for statistical analysis **** *p* < 0.0001, and ns = not significant.

The phosphorylation of Akt/mTOR and NF-κB signaling pathways are found to be constitutively activated in TNBC cells. The activation of such pathways is associated with increased cell survival, prevention of cell cycle arrest, and resistance to apoptosis [10,14,17]. To further investigate the mechanism leading to cell death and cell cycle arrest in TNBC by C828, we examined the effect of C828 in the inhibition of these pathways. To do so, we collected whole cell lysates of SUM159PT, MCF7, and MCF10A cells after treatment with C828 for 24 h to Western blot for the detection of key proteins in both pathways. We found that there was a decline in Akt phosphorylation at Ser473 and Thr308 and mTOR at S2448 in SUM159PT cells when treated with five µM of C828. Similarly, a decrease in phosphorylation of p65 (Ser536) was observed at five µM treatment (Figure 5A). However, in non-TNBC cells (MCF7 and MCF10A), no observed changes in phosphorylation of Akt/mTOR and NF-κB signaling pathways were found (Figure 5B,C). Thus, C828 inhibited phosphorylation of Akt/mTOR and NF-κB signaling pathways in TNBC cells while non-TNBC cells remained unaffected. 

### 2.8. Aurantoside C Activates Phosphorylation of p38 MAPK and SAPK/JNK Signaling Pathways in TNBC Cells 

**Figure 6:**
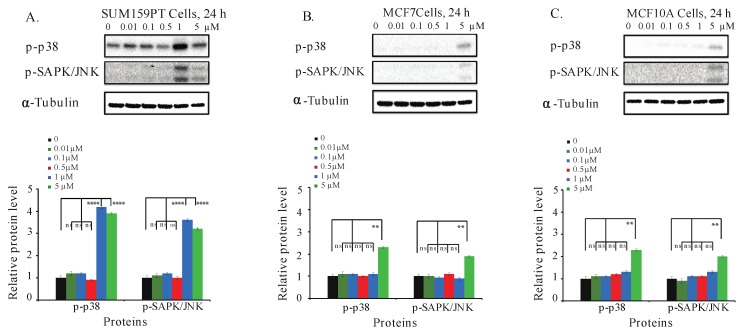
C828 activates phosphorylation of MAPK pathways in TNBC cells. (**A**) SUM159PT cells, (**B**) MCF7 cells, and (**C**) MCF10A cells were treated with 0, 0.01, 0.1, 0.5, 1, and 5 μM of C828 for 24 h. Whole cell lysates were isolated, quantification of proteins was done and immunoblotted with antibodies specific for p-p38 MAPK and p-SAPK/JNK. α-tubulin was used as a loading control for each experiment. ImageJ was used to quantify each blot. The blots were normalized against tubulin and then against control protein (when C828 was not added). Two independent experiments were performed. The histogram below each western blot represents the average normalized values of the band from each experiment. One way ANOVA with Tukey’s post hoc test was used for statistical analysis **** *p* < 0.0001, ** *p* < 0.01, and ns = not significant.

Many anticancer compounds activate SAPK/JNK and p38 MAPK, inhibiting proliferation and causing apoptosis of cancer cells [49,50,51]. Thus, we next investigated the effect of C828 in phosphorylation of p38 and SAPK/JNK signaling pathways in TNBC and non-TNBC cells. We found that both one and five µM treatments increased the phosphorylation of p38 MAPK and SAPK/JNK in SUM159PT cells (Figure 6A). The increase in phosphorylation of p38 MAPK and p-SAPK/JNK in MCF7 and MCF10A cells was only observed at higher concentrations, i.e., five µM (Figure 6B,C). These results suggest that the phosphorylation of p38 MAPK and SAPK/JNK pathways were upregulated after the treatment with C828 in TNBC cells, leaving non-TNBC cells unaffected. 

### 2.9. Aurantoside C Triggers Apoptosis in TNBC Cells

**Figure 7:**
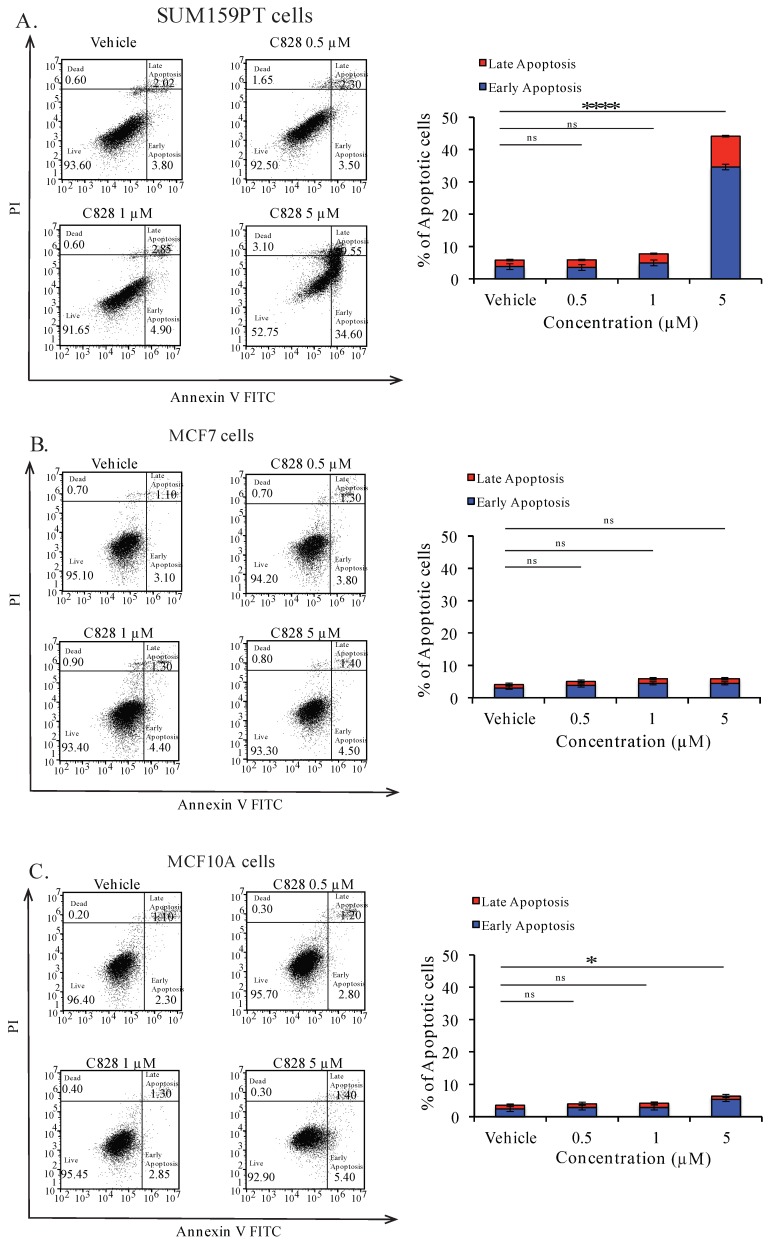
C828 induces apoptotic cell death in TNBC cells. (**A**) SUM159PT cells, (**B**) MCF7 cells, and (**C**) MCF10A cells were treated with vehicle control and 0.5 μM, 1 μM, and 5 μM of C828, respectively, for 24 h. Treated cells were stained using Annexin-V FITC and PI and analyzed by flow cytometry and data were processed using Flow Jo 10. Experiments were performed in triplicates and the bar diagram represents the percentage of apoptotic cells (early and late apoptosis appearing in the right lower and upper quadrants). One way ANOVA with Tukey’s post hoc test was used for statistical analysis **** *p* < 0.0001, * *p* < 0.1, and ns = not significant.

Apoptosis is a programmed cell death that occurs during development and aging as a homeostatic event to maintain cell populations [52,53]. Deregulation in programmed cell death leads to proliferation and restoration of such apoptotic pathways could effectively treat malignancy [54]. Here, we investigated if there was induction of apoptosis in TNBC cells when treated with C828. To do so, SUM159PT, MCF7, and MCF10A cells were treated with 0.5 μM, one μM, and five μM of C828 for 24 h and stained using Annexin-V FITC and Propidium Iodide. The apoptotic cell population were determined by flow cytometry. We found that the total percentage of cells (early and late apoptotic population) significantly increased from 6% in vehicle control group to 45% when treated with five μM of C828 in SUM159PT cells (Figure 7A). Treatment with 0.5 μM and 1 μM caused no change in apoptotic cells population in SUM159PT cells (Figure 7A). In MCF7 and MCF10A cells, no change was observed when treated with 0.5 μM and one μM of C828. However, five μM treatment caused slight increase in apoptotic cell population from 4% in vehicle control group to 6% in MCF7 cells and from 3% in vehicle control group to 7% in MCF10A cells (Figure 7B,C). Thus, these results showed that C828 induced significant increase in apoptotic cell population in TNBC cells, whereas in non-TNBC cells, a minor increment in apoptotic cell population was observed.

**Figure 8:**
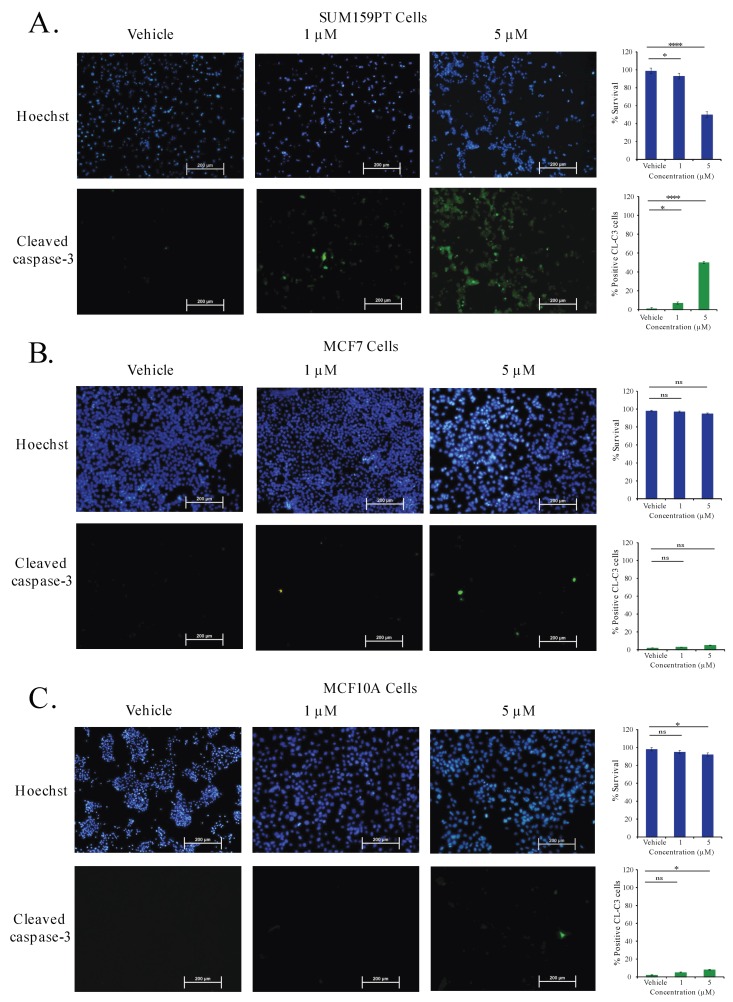
C828 selectively induces apoptotic cell death in TNBC cells. (**A**) SUM159PT cells, (**B**) MCF7 cells, and (**C**) MCF10A cells were treated for 24 h with vehicle control and 0.5 μM and 1 μM of C828 respectively and analysed by immunofluorescence assay. Cells were stained with anti-cleaved caspase-3 antibody (green) and counterstained with Hoechst 33258 (blue). Image J software was used to quantify the cells that are shown in bar diagram. Experiments were performed in triplicates. One way ANOVA with Tukey’s post hoc test was used for statistical analysis **** *p* < 0.0001, * *p* < 0.1, and ns = not significant.

To further verify the effect of C828 in inducing apoptosis in TNBC and non-TNBC cells, SUM159PT, MCF7, and MCF10A cells were treated with vehicle control, one μM and five μM of C828 for 24 h and apoptotic cell death was determined by immunofluorescence. We found that in SUM159PT cells, there was an increment in apoptotic cells from 2% in vehicle control cells to 7% and 50% when treated with one and five μM of C828, respectively (Figure 8A). No significant changes in apoptotic cell population were seen in MCF7 and MCF10A cells when treated with one μM (Figure 8B,C). At five μM treatment, the apoptotic cell population increased slightly from 2% in vehicle control group to 5% in MCF7 cells (Figure 8B) and 2% in vehicle control group to 8% in MCF10A cells (Figure 8C). These result show that C828 induced apoptosis in TNBC cells while non-TNBC cells were affected to comparatively lower extent.

## 3. Discussion

The evaluation of natural products in drug discovery has a long history of providing novel compounds for the treatment of human disease [28,55]. In this study, we show that a metabolite, Aurantoside C (C828), derived from the marine sponge collected off the coast of WA, *M*. *lynbeazleyae* could be a promising anticancer agent for treating TNBC. Aurantoside C was first isolated from the sponge *Homophymia conferta* (now known as *Manihinea conferta*) [38]. To date, 11 different aurantosides (A to K) have been isolated from different marine sponges [38,56,57,58,59,60]. While Aurantosides A and B have shown cytotoxic activity against leukemia cells [56], Aurantosides D to K have failed to show any cytotoxic activities in cancer cells [57,58,59,60]. However, Aurantoside C (C828) has not yet been tested for cytotoxic activity in any cancer study. Of note, our study is the first to demonstrate the cytotoxic effect of C828 in cancer cells and in particular, TNBC cells. 

TNBC, lacking ER, PR, and HER2 receptors, found in other breast cancer subtypes, cannot be treated with chemotherapeutic drugs that target these receptors [61]. Hence, standard chemotherapy such as platinum compounds, anthracyclines, and taxanes are often the treatment of choice for these patients [39]. One of the drawbacks of the chemotherapeutic drugs used currently in clinics to treat TNBC is the lack of selectivity and specificity. Hence, it is essential to develop drugs that can selectively target TNBC cells. In this study, we found that C828 reduced cell viability of TNBC cells at very low micro-molar concentrations, while higher doses were required to exhibit cytotoxicity in less aggressive breast cancer cells and normal epithelial cells of the breast. Additionally, when we compared the effect of C828 in reducing cell viability of TNBC cells with chemotherapeutic drug doxorubicin and cisplatin, we found that C828 was 20 and 35 times more effective in comparison to doxorubicin and cisplatin respectively. Thus, C828 represents a highly active cytotoxic compound operating at very low micro-molar concentrations in contrast to other chemotherapeutic drugs tested in vitro for TNBC, including the most targeted therapeutic agents for breast cancer such as cetuximab or erlotinib [62,63,64]. 

In this work, we also demonstrate that C828 induced accumulation of cells at S-phase in TNBC. It is well known that cell cycle progression plays an important role in proliferation of cancer cells and its arrest can potentially control the growth of cancer cells [45]. Cyclins and cyclin dependent kinases (CDK) positively influence the cell cycle progression, and cyclin dependent kinase inhibitors (CDKIs) participate in important cell cycle regulatory checkpoints promoting cell cycle arrest [46]. A sequential activation and degradation of cyclins and CDK is essential for the progression through the cell cycle. Cyclin D1 and CDK4/6 participate in the early G1 phase of cell cycle and cyclins E1 take part in the transition G1/S [65,66]. At the S-phase, there is a degradation of cyclin D1, cyclin E1, CDK4, and CDK6 as well as an increase in other cyclins and CDKs types [65,66]. Our results showed that treatment with C828 resulted in significant accumulation of cells in S-phase correlating with decrease in number of cells in G1 phase. This accumulation of cells at the S-phase of cell cycle was concomitant with a decrease in cyclin D1, CDK4, CDK6, cyclin E1, and an increase of the expression of the CDKI, p21. Those changes were exclusive to TNBC cells as no changes in cyclins and p21 expression were observed in non-TNBC cells. Similar results were reported for Hinokitol, a tropolone-related natural compound that causes S-phase arrest of human colon cancer cells mediated by increased expression of p21 [67].

Besides the cell cycle arrest, it was seen that C828 inhibited the phosphorylation of Akt/mTOR and NF-κB signaling pathways in TNBC. The ability of C828 in inhibiting the phosphorylation of Akt/mTOR and NF-κB pathways is particularly relevant as TNBC has constitutive activation of these pathways [10,11,17], which favors metastatic processes and chemo-resistance mechanisms [16,18]. Our result are in agreement with those reported for stellettin B, a triterpene that was derived from marine sponge *Jaspis stellifera* inhibited proliferation and induced cell death in human non-small cell lung cancer, human chronic myeloid leukemia and human glioblastoma cancer cells by inhibiting Akt/mTOR signaling pathways [68,69,70]. Moreover, it is known that the upregulation of Akt and NF-κB signaling pathways is related to the activation of cyclin D1 that leads to proliferation of cancer cells [21,71] and decline in CDKI, p21, thus inhibiting apoptosis [72,73,74,75,76,77]. In this study, the inhibition of NF-κB and Akt/mTOR pathways in conjunction with a decline in cyclin D1 suggests that cyclin D1 is a direct downstream target of these pathways. Thus, these results suggest that the inhibition of phosphorylation of both Akt/mTOR and NF-κB signaling pathways together with the decrease in cyclin D1 and increase in p21 leads to the accumulation of the cells at S-phase and hence, decrease in cell viability seen in the TNBC cell lines. Importantly, none of these effects in the signaling pathways were observed in luminal breast cancer cells or in normal-like breast epithelial cells, thus highlighting the potential of C828 as a therapeutic candidate with an enhanced sensitivity for TNBC cells.

MAPK signaling pathways also play a major role in cell proliferation, survival and response to stress in TNBC [23]. Similarly, apoptosis plays a vital role in cancer cells and is regulated by the cleavage of the caspase-3 protein [52]. Cleaved caspase-3 then executes programmed cell death [54]. It was found that the activation of phosphorylation of stress related MAPK pathways, SAPK/JNK and p38 MAPK induces apoptosis through cleavage of caspase-3 [50]. In this study, we observed that C828 upregulated the phosphorylation of p38 MAPK and SAPK/JNK pathways and also triggered apoptosis in TNBC cells. Our result is in agreement with previous reports such as *Physalis angulata* L., a traditional Chinese herb medicine, which has been shown to activate JNK and induce apoptosis and autophagy in TNBC cells [78]. Similarly, isoliensinine, an alkaloid found in the seed embryo of lotus (*Nelumbo nucifera*) induced apoptosis in TNBC through a similar mechanism [79]. Moreover, it has also been reported that the activation of SAPK/JNK and p38 MAPK stabilizes CDKI, p21 that is responsible for cell cycle arrest [80]. Thus, the activation of phosphorylation of p38 MAPK and SAPK/JNK pathways together with upregulation in p21 suggests that the upregulation of these signaling pathways leads to arrest of the TNBC cells that consequently leads to apoptosis in TNBC cells. 

It is to be noted that this study is focused in a 24 h time frame. However, the recent review by EORTC-PAMM group discusses that some drugs, such as cisplatin forms mono-adduct with DNA in a shorter exposure time (1 h) [81]. In future, it would be interesting to pursue this line of investigation and present a detailed comparative study of the viability/cytotoxicity at different time intervals. Additionally, with an ultimate aim of drug development, further studies regarding in vivo activity of C828 in TNBC cells will be performed in the future.

## 4. Materials and Methods 

### 4.1. General Experimental Procedures 

Bruker Avance 500 MHz spectrometers (Bruker Corporation, Karlsruhe, Germany) were used to record all NMR spectra using methanol-d_4_ as solvent. Chemical shifts were reported with reference to the residual solvent peak δ_H_ 3.31 and δ_C_ 49.0 ppm. Waters LCT Premier XE (Waters, Sydney, NSW, Australia) time-of-flight mass spectrometer was used for high-resolution mass spectra in positive electrospray ionization mode. Apollo reversed phase C18 column (250 mm × 10 mm, 5 μM, Grace-Davison Discovery Sciences, Melbourne, VIC, Australia) was used for separation in semi-preparative HPLC (Agilent Series 1200). HPLC was equipped with a photodiode array detector and preparative fraction collector.

### 4.2. Reagents

Unless otherwise stated, all reagents were purchased from Sigma-Aldrich (St. Louis, MO, USA). All solvents used were of HPLC grade.

### 4.3. Details of Sponge Materials and Identification

The sponge *Manihinea lynbeazleyae* (museum registration number Z31539) was collected in 2005 at Perth Canyon, Western Australia at the depth of 232 m, and has been stored wet frozen.

### 4.4. Purification and Isolation of Aurantoside C (C828)

The bright red wet sponge (66.4 g) was crushed, grinded using mortar and pestle and extracted overnight with 1:1 dichloromethane (DCM) and methanol. The red crude extracts were filtered, combined and solvent was evaporated to yield a red solid (5.8 g). Flash chromatography using silica gel (Grace Davisil^®^, LC60A, particle size 40–63 μm) was used to separate the crude solid extract (5 g). The column was eluted with solvents starting with 100% hexanes to 100% ethyl acetate to 100% methanol (gradient elution) to obtain six different fractions. These fractions were tested for their cytotoxic activities in SUM159PT cells for 24 h (Cell Titer Glo^®^ assay). Testing of a sample of each fraction (0.1 mg/mL) fractions showed activity was present in fraction 6, corresponding to 100% methanol elution. The active fraction 6 (1.8 g) was further subjected to high-pressure liquid chromatography (HPLC) with an isocratic solvent system of 55% (*v*/*v*) acetonitrile/water (+0.1% TFA). A total of 9 fractions were collected and tested in SUM159PT cells. The fraction collected between 17–19 min was found to be the most active. Solvent was evaporated to yield aurantoside C (C828) as a bright red solid (0.015 g, 0.02%, based on the wet weight of the sponge) that was active in SUM159PT cells. Spectroscopic data presented below are in agreement with one reported for C828 [38] and for Aurantoside A and B [56]. 

**Aurantoside C**: red amorphous solid, IR (neat) ν_max_ 3321, 2943, 2832, 1672, 1485, 1202, 1135, 1049, 800 cm^−1^; ^1^H NMR (CD_3_OD, 500 MHz) δ 4.29 (1H, br, H-4), 2.67(1H, br, d, *J* = 16 Hz, H-5a), 2.78 (1H, dd, *J* = 4 Hz, 16 Hz, H-5b), 7.23 (1H, br, d, *J* = 12 Hz, H-8), 7.62 (1H, m, H-9), 6.65 (1H, m, H-10), 6.88 (1H, dd, *J* = 11.5 Hz, *J* = 14.6 Hz, H-11), 6.52 (1H, dd, *J* = 11.5 Hz, *J* = 14.5 Hz, H-12), 6.74(1H, m, H-13), 6.71(1H, m, H-14), 6.66(1H, m, H-15), 6.76 (1H, d, *J* = 11 Hz, H-16), 6.53 (1H, d, *J* = 14.5 Hz, H-18), 6.96(1H, dd, *J* = 10.5 Hz, *J* = 14.5 Hz, H-19), 6.35 (1H, d, *J* = 10.5 Hz, H-20), 2.23 (3H, s, H-22) 4.46 (1H, br, H-1′), 4.48 (1H, br, H-2′), 3.47 (1H, t, *J* = 9 Hz, H-3′), 3.88 (1H, ddd, *J* = 5 Hz, *J* = 9 Hz, *J* = 10 Hz, H-4′), 3.21 (1H, t, *J* = 11 Hz, H-5′a), 3.89 (1H, m, H-5′b), 5.03 (1H, br, H-1″), 3.89 (1H, m, H-2″), 3.60 (1H, m, H-3″), 3.77 (1H, m, H-4″), 3.62 (1H, m, H-5″a), 3.75 (1H, m, H-5″b), 4.92 (1H, d, *J* = 4.5 Hz, H-1‴), 3.92 (1H, m, H-2‴), 3.92 (1H, m, H-3‴), 3.88 (1H, m, H-4‴), 1.31 (3H, d, *J* = 6 Hz, H-5‴). ^13^C NMR (CD_3_OD, 500 MHz) δ 174.3 (CH, C-1), 145.4 (CH, C-9), 137.4 (CH, C-21), 135.6 (CH, C-12), 135.4 (C, C-14), 133.3(CH, C-10), 132.7 (C, C-19), 132.0(CH, C-15), 131.4 (CH, C-16), 128.9 (CH, C-17), 126.1 (CH, C-20), 121.9 (CH, C-8), 103.8 (CH, C-1″), 100.6 (CH, C-1‴), 81.0 (C, C-1′), 79.4 (CH, C-3‴), 79.2 (CH, C-4‴), 78.8 (C, C-2′), 76.4 (CH, C-4″), 71.6 (CH, C-2″), 70.7 (CH, C-4′), 70.4 (CH, C-3″), 69.1 (CH_2_, C-5′), 61.8 (CH_2_, C-5″), 59.6 (CH, C-3′), 38.0 (CH_2_, C-5), 26.7 (CH, C-22), 20.7 (CH_3,_ C-5‴). HRESIMS *m*/*z* 829.6216 [M + H]^+^ (calcd. for C_37_H_47_N_2_O_15_Cl_2_, 829.6218).

### 4.5. Cell Culture

Human cell lines MCF7, SUM159PT, and SUM149PT were received from the Tissue Culture Facility of the UNC Lineberger Comprehensive Cancer Centre (University of North Carolina, Chapel Hill, NC, USA). MDA-MB-231, T47D, MCF10A, and ZR-75-1 cell lines were obtained from the American Type Culture Collection (Manassas; VA, USA). ZR-75-1 and T47D cells were cultured and grown in RPMI medium. SUM159PT and SUM149PT cells were cultured and grown in DMEM-F/12 (Life Technologies, Melbourne, VIC, Australia) media. MDA-MB-231 cells were cultured in DMEM (Life Technologies, VIC, Australia) media. MCF7 cells were cultured and grown in MEM alpha. All media were supplemented with 10% fetal bovine serum (Life Technologies) and 1% penicillin/streptomycin (Life Technologies) except SUM159PT media that was supplemented with 5% fetal bovine serum. MCF7 media was supplemented with 1% sodium bicarbonate (100 mM), 1% sodium pyruvate (7.5 mM), and 1% MEM non-essential amino acid (100×) (Life Technologies). SUM159PT media was supplemented with 5 μg/mL insulin and 1 μg/mL hydrocortisone. MCF10A were cultured in a DMEM-F/12 supplemented with 10% horse serum, 20 ng/mL EGF, 0.5 μg/mL hydrocortisone, 10 mg/mL insulin, 10 ng/mL cholera toxin, and 1% penicillin/streptomycin (Life Technologies). Cell lines were cultured in 10 cm^2^ petri-dish (Corning) and were maintained in humidified incubators at 37 °C with 5% CO_2_. Cells were passaged at 80% confluency, and media changed every 4–5 days.

### 4.6. Cell Viability Assay

SUM159PT and MDA-MB-231 cells (5000 cells/well), SUM149PT, MCF7, and T47D, (7000 cells/well) and ZR-75-1, MCF10A, and MCF12A (8000 cells/well) were cultured in 96-well white plate and left overnight to adhere. C828 was dissolved in 0.1% DMSO and diluted with media to get the required concentrations. Cells were treated with C828 for 24 h. Cell Titer Glo^®^ was used to detect cell viability according to manufacturer’s protocol (Promega, Sydney, NSW, Australia) and luminescence was measured using the Envision 2012 Multi-label Reader (PerkinElmer, Waltham, MA, USA). Graphpad Prism 6 was used to calculate IC_50_ of C828.

### 4.7. Cell Cycle Analysis

Cells (500,000 cells/well) were cultured in 6 well plates left to adhere overnight. To evaluate the effect of C828 on cell cycle distribution, cells were treated with different concentrations of C828 for 24 h. Cells were collected, washed twice with FACS buffer, and fixed with 70% ethanol overnight at −20 °C. The next day, cells were washed with ice-cold PBS and incubated with RNase A (50 μg/mL, Qiagen Kit (Melbourne, VIC, Australia) for half an hour at 37 °C. Fixed cells were then stained with Propidium iodide (50 μg/mL, Sigma Aldrich, St. Louis, MO, USA) in dark for half an hour at room temperature and analyzed using flow cytometry (BD Accuri C6). FlowJo 10 (FlowJo LLC, Ashland, OR, USA) was used to process the data.

### 4.8. Western Blot Analysis

Cells (1,000,000 cells/well) were cultured in 6 well plates and left to adhere overnight and then treated with different concentration of C828 at various time points. Treated cells were detached, collected, and lysed on ice for 5 min in lysis buffer (400 mM NaCl, 0.5% triton X-100, 50 mM tris pH 7.4) and sonicated for 40 seconds at 10 mA. Quantification of proteins were done and proteins were separated in 4–15% SDS-PAGE (BioRad, Sydney, NSW, Australia) and transferred electrically onto PVDF membrane (BioRad, Sydney, NSW, Australia). Blocking of the membrane was carried out with 5% skim milk powder in TBST, washed three times with TBST and incubated with specific primary antibodies overnight at 4 °C. Primary antibodies used were cyclin D1, cyclin E1, CDK4, CDK6, CDK2, p21, p-Akt (Ser473), p-Akt (Thr308), p-mTOR (Ser2448), p-p38 MAPK, p-SAPK/JNK, and p-p65 (Ser536) (Cell Signaling Technologies, Brisbane, QLD, Australia) that were diluted following manufacturer’s protocol. Secondary antibodies used were horseradish peroxidase (HRP)-conjugated secondary antibodies (1:10,000 dilution, GE Technologies, Brisbane, QLD, Australia). Membranes were incubated with secondary antibodies for 1 h at room temperature. Detection of the proteins were carried out using Clarity Western ECL detection kit (BioRad, Sydney, NSW, Australia). α-Tubulin (monoclonal, 1:5000, clone B512, Sigma-Aldrich, St. Louis, MO, USA) was used as a loading control.

### 4.9. Apoptosis Assay Using Annexin-V-PI-Binding Assay

Cells (500,000 cells/well) were cultured in 6 well plated and left to adhere overnight. After 24 h, treatment of cells with different concentrations of C828 was carried out. Cells were treated for a further 24 h. Annexin-V/FITC Apoptosis Detection kit was used to detect the population of apoptotic cells according to manufacturer’s protocol (BD Bioscience, Perth, WA, Australia). In brief, after 24 h of treatment, cells were collected, washed two times with cold PBS, and dissolved in 1× binding buffer. Cells were spinned and approximately 100,000 cells were collected and stained using Annexin-V/FITC and Propidium iodide staining kit at room temperature for 15 min in the dark, and analyzed by flow cytometry (BD Accuri C6, Sydney, NSW, Australia). Analysis of the result was carried out using FlowJo 10 (FLowJo LLC, Ashland, OR, USA).

### 4.10. Apoptosis Assay by Immunofluorescence

Cells (40,000 cells/well) were cultured in 24 well plated in coverslips that were pre-coated with poly-lysine and left to adhere overnight. Treatment of cells with C828 was carried out for 24 h. In brief, the next day, cells were fixed at room temperature with 4% paraformaldehyde for 20 min, washed with PBS, blocked with blocking solution (3% BSA in PBS). Fixed cells were incubated with primary antibody (anti-cleaved caspase-3, 1:500 dilution, Cell Signaling Technology, QLD, Australia) overnight at 4 °C. The following day, the cells were washed with PBS and incubated with an anti-rabbit secondary antibody Alexa Fluoro 488-conjugated antibody (1:5000 dilution; Cell Signaling Technologies, QLD, Australia) and Hoechst 33258 (1:10,000 dilution) nuclei for 1 h at room temperature. The coverslips were washed with PBS and mounted on slides. Olympus IX71 microscope (Melbourne, VIC, Australia) was used to record the fluorescent images.

### 4.11. Statistical Analysis

GraphPad Prism version 6 (GraphPad Software Inc., La Jolla, CA, USA) was used to analyze statistics using an unpaired one-way ANOVA with the Tukey’s post hoc test correcting for multiple comparisons.

## 5. Conclusions

In conclusion, we have isolated aurantoside C (C828) from the marine sponge *M*. *lynbeazleyae* collected from Western Australia using bioassay guided fractionation. Aurantoside C has shown increased sensitivity towards TNBC cells over non-TNBC cells at a very low micromolar concentration. We have further explored the mechanism of action of C828 and showed it causes S-phase accumulation of SUM159PT cells, decreases the cyclins and cyclin dependent kinases, and increases the cyclin dependent kinase inhibitor, p21 in TNBC cells. Treatment with C828 also resulted in inhibition decline of phosphorylation of Akt/mTOR and NF-κB signaling pathways, activation of phosphorylation of SAPK/JNK and p38 MAPK signaling pathways, causing apoptosis in TNBC cells, while non-TNBC cells (MCF7 and MCF10A) cells remained almost unaffected. Altogether, these results indicate that C828 could be considered as a targeted therapeutic agent for TNBC cells and at the same time unveil unknown cell signaling mechanisms in inhibiting cell growth and induction of cell death triggered by this compound. With an ultimate aim of drug development, further studies regarding in vivo activity of C828 in TNBC cells will be performed in the future.

## Figures and Tables

**Table 1:** Comparison of C828 with C800 in TNBC cells.

Compounds	Cell Lines	IC_50_ (µM)
C828	SUM159PT	0.56 ± 0.01
	MDA-MB-231	0.61 ± 0.01
	SUM149PT	0.81 ± 0.02
C800	SUM159PT	3.42 ± 0.07
	MDA-MB-231	5.00 ± 0.57
	SUM149PT	6.02 ± 0.11

## Supplementary Materials

The following are available online at http://www.mdpi.com/1660-3397/16/10/361/s1, Figures S1 and S2: ^1^H and ^13^C NMR spectra of isolated Aurantoside C.
