# Supplementary material for: Aurantoside C Targets and Induces Apoptosis in Triple Negative Breast Cancer Cells
Source: Mar Drugs. 2018 Oct 1;16(10):361. doi: 10.3390/md16100361 (PMC6213655; doi:10.3390/md16100361)

marine drugs

Article

Aurantoside C targets and induces apoptosis in Triple Negative Breast Cancer cells

Sumi Shrestha[1,2], Anabel Sorolla[2], Jane Fromont[3], Pilar Blancafort[2]*, and Gavin R. Flematti[1]*

[1] School of Molecular Sciences, The University of Western Australia, Crawley, Western Australia, 6009, Australia;
[2] Cancer Epigenetics, Harry Perkins Institute of Medical Research, QEII Medical Centre and Centre for Medical Research, The University of Western Australia, Crawley, Western Australia, 6009, Australia;
[3] Western Australian Museum, Welshpool, Western Australia 6106, Australia;
* Correspondence: gavin.flematti@uwa.edu.au; Tel.: +61 6488 4461 (Chemistry) (G.R.F) pilar.blancafort@uwa.edu.au; Tel.: +61 8615 1099 (Cancer Biology) (P.B)

Received: date; Accepted: date; Published: date

Contents:

S1. ^{1}H NMR of Aurantoside C (C828) in CD_3OD (500MHz)
S2. ^{13}C NMR of. Aurantoside C (C828) in CD_3OD (500MHz)
S3. Cell viability of C828 in A. SUM159PT cells, B. MCF7 cells, and C. MCF10A cells at 24 h, 48 h and 72 h

S1. ^{1}H NMR of Aurantoside C (C828) in CD_3OD (500MHz)

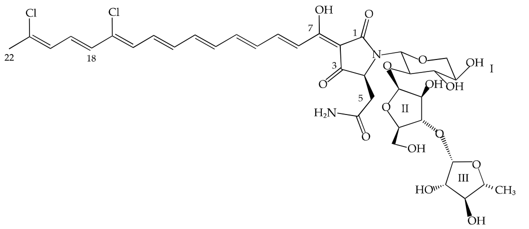

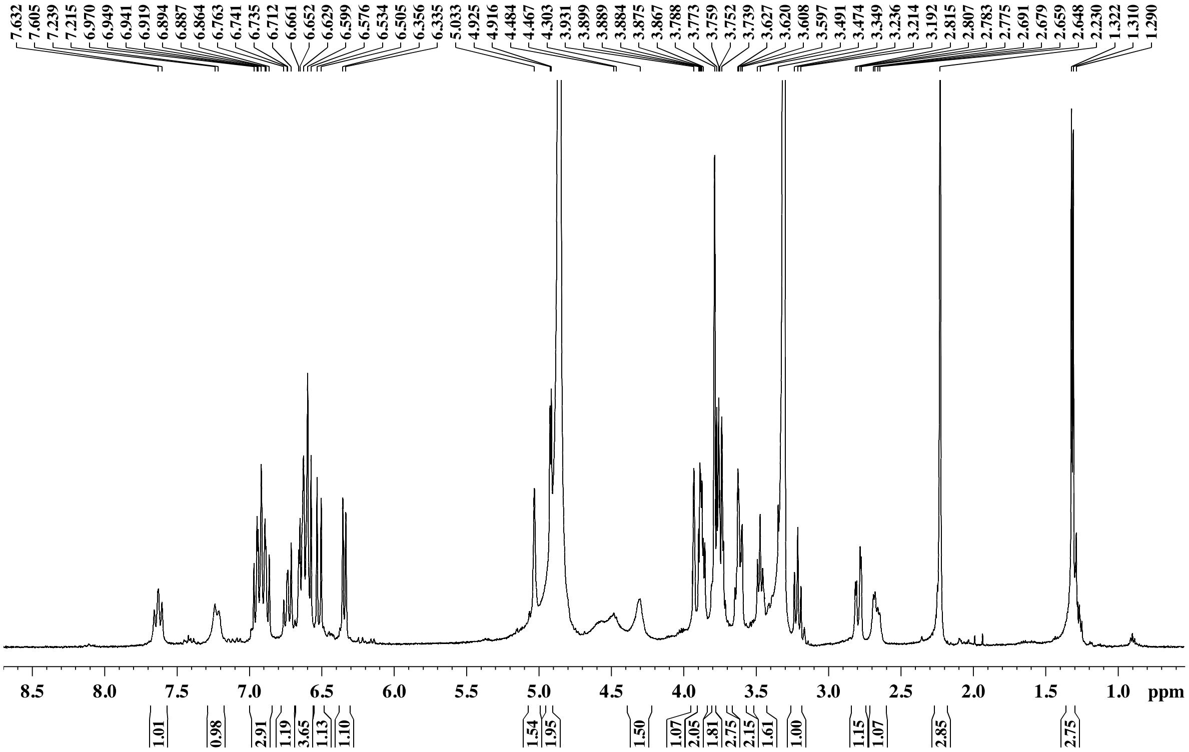

S2. ^{13}C NMR of Aurantoside C (C828) in CD_3OD (500MHz)

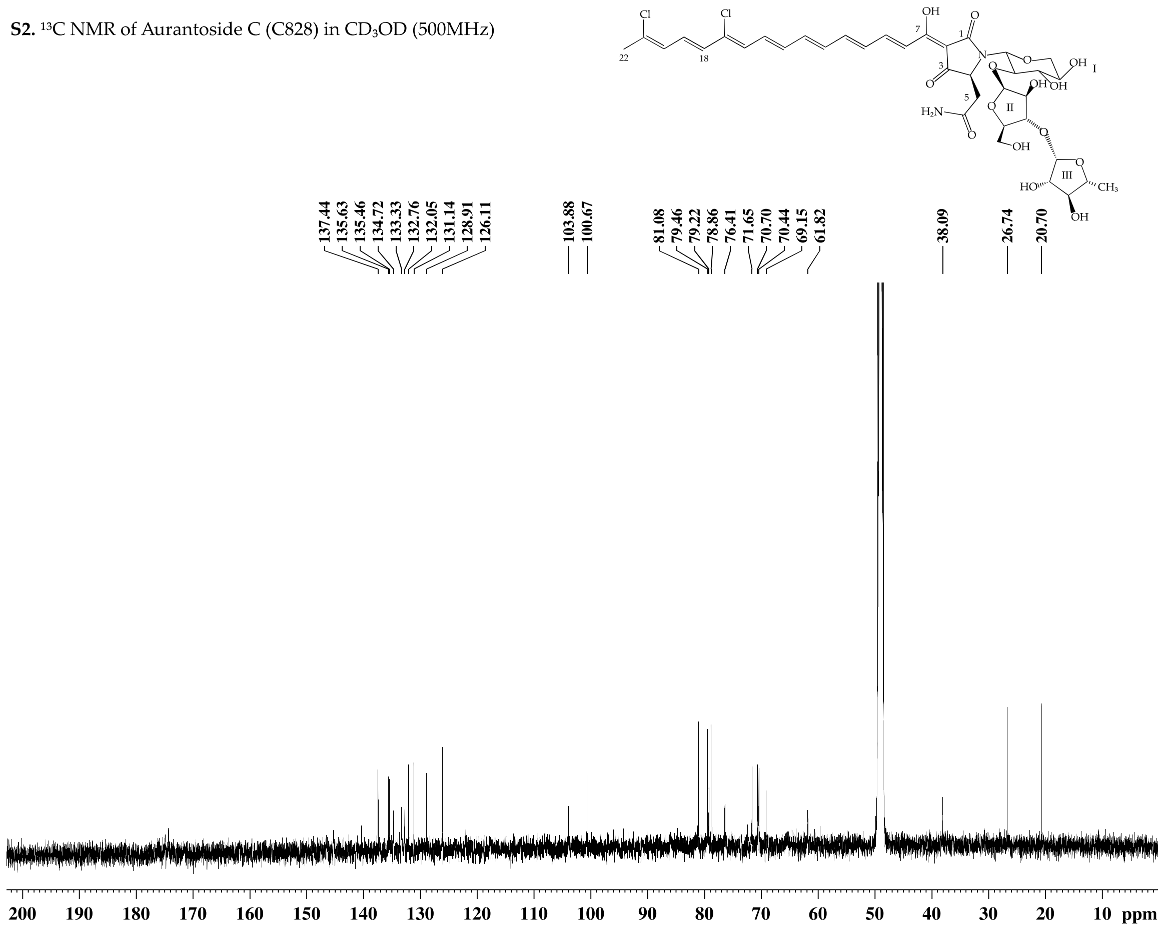

S3. Cell viability of C828 in A. SUM159PT cells, B. MCF7 cells, and C. MCF10A cells at 24 h, 48 h and 72 h

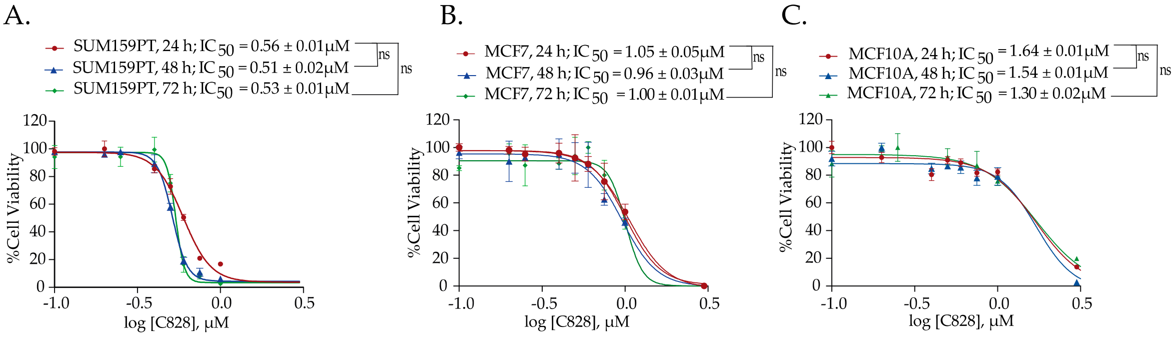

Supplement: Supplementary file 1 [file marinedrugs-16-00361-s001.pdf]
